# Fasta2Structure: a user-friendly tool for converting multiple aligned FASTA files to STRUCTURE format

**DOI:** 10.1186/s12859-024-05697-7

**Published:** 2024-02-15

**Authors:** Adam Bessa-Silva

**Affiliations:** Laboratório de Evolução, Universidade Federal do Pará, Alameda Leandro Ribeiro, Aldeia, Bragança, Pará Brazil

**Keywords:** STRUCTURE format, Multiple FASTA files, Tkinter, Biopython, Aligned sequences, Population genetics

## Abstract

**Background:**

The STRUCTURE software has gained popularity as a tool for population structure and genetic analysis. Nevertheless, formatting data to meet STRUCTURE's specific requirements can be daunting and susceptible to errors, especially when handling multilocus data. This article highlights the creation of a graphical user interface (GUI) application tailored to streamline the process of converting multiple sequence alignments into a single, cohesive file that is compatible with the STRUCTURE software.

**Results:**

The application has been developed utilizing Tkinter for the GUI and Biopython for handling FASTA files. This program processes the files, pinpoints variable sites, and converts the sequences into a binary format. Subsequently, the sequences are concatenated and presented within the graphical interface's text area, enabling users to review and confirm the results. Furthermore, the program stores the concatenated results in a file, delivering a ready-to-use input for the STRUCTURE software.

**Conclusion:**

This application offers an efficient and dependable solution for transforming multiple aligned FASTA files into a concatenated binary format file, which is compatible with the STRUCTURE software. With its user-friendly graphical interface and error-reduction approach, this tool proves invaluable for researchers engaged in population structure and genetic analysis.

## Background

Population structure analysis is a vital aspect of understanding genetic diversity within and between populations, providing insights into the evolutionary history of species and facilitating various applications in ecology, conservation, and breeding [1, 2]. The population structure can be inferred from molecular markers with diverse characteristics, encompassing data derived from multiple genes, both mitochondrial and nuclear [3, 4].

In this context, The STRUCTURE software, developed by Pritchard et al. [5], has emerged as a popular tool for inferring population structure from multilocus data. It employs a Bayesian model-based clustering algorithm to assign individuals to populations based on their genotypes, allowing researchers to identify genetically distinct populations and admixed individuals [5]. This software has been extensively used in population genetics, conservation biology, and breeding programs, as well as in various fields of ecology and evolutionary biology [6, 7].

Despite the widespread adoption of STRUCTURE in population genetics research, the preparation of data in the specific format required by the software can be both laborious and error-prone, particularly when handling multiple aligned sequence files. Researchers often need to manipulate and concatenate their sequence data to generate input files that are compatible with STRUCTURE, which can result in inaccuracies and inconsistencies if not conducted meticulously [8]. Furthermore, this process can be time-consuming and might necessitate advanced knowledge of programming languages or scripting skills [9]. Additionally, the rapid advancements in sequencing technologies have facilitated the acquisition of multilocus data for population genetics studies, creating a substantial demand for user-friendly tools to convert and manipulate data, including those tailored for population structure analyses.

In response to these challenges, we have developed a graphical user interface (GUI) application designed to transform multiple aligned FASTA files into a single concatenated format file suitable for use with the STRUCTURE software. This application aims to streamline the data preparation process, thereby minimizing the potential for errors and making the task more accessible to researchers with limited programming experience. By offering an intuitive and efficient solution, we endeavour to accommodate the growing demand for data conversion and manipulation tools within the realm of population genetics research, ultimately enhancing the overall accessibility and reproducibility of population structure analyses.

## Implementation

The development of the software tool consists of several steps, aimed at identifying and concatenating the variable positions of each sequence. The tool employs the tkinter library for the graphical interface, the BioPython library for FASTA file processing, and the os, threading, and traceback libraries for file manipulation and missing data. The construction of the tool was divided into the following steps:

1. Graphical interface and file selection
  - Development of the graphical interface using the tkinter library, creating a window (root) for the application.
  - Implementation of a button (browse_button) that, when clicked, triggers the browse_files function.
  - The browse_files function uses the filedialog.askopenfilenames function to allow the user to select multiple FASTA files.
2. Reading and processing FASTA files
  - In the browse_files function, for each selected file, the process_fasta_file function is called with the arguments: filepath (file path), sequence_dict (dictionary to store sequences), file_count (total number of files), and progress_callback (function to update the progress bar and progress label).
  - The process_fasta_file function uses the AlignIO.read function from the BioPython library to read the sequence alignment from the FASTA file.
3. Identification of variable positions
  - The get_variable_sites function is called within the process_fasta_file function, receiving the alignment as an argument.
  - The get_variable_sites function iterates through each column of the alignment and identifies variable positions, adding the column index to a list (variable_sites) that is returned at the end.
4. Conversion of sequences to binary format
  - The convert_to_binary function is called within the process_fasta_file function, receiving a variable positions sequence as an argument.
  - The convert_to_binary function maps the characters ‘A’, ‘T’, ‘C’, and ‘G’ to the values ‘0’, ‘1’, ‘2’, and ‘3’, respectively, and the characters ‘−‘ and ‘?’ to the value ‘− 9’, converting the variable positions sequence into a binary sequence.
5. Storage and concatenation of sequences
  - In the process_fasta_file function, the binary sequences are stored in the sequence_dict dictionary, using the sequence identifier as the key and a list containing the binary sequence and file count as values.
  - The pad_missing_sequences function is called after processing all files, filling in the gaps of sequences do not present in all files, adding the value ‘− 9’ in the missing variable positions.
  - The convert_to_binary(sequence) function iterates over each base in the input sequence, converting it to its corresponding binary value using the binary_mapping dictionary. In the case of indels, they are mapped to ‘− 9’ due to the limitations of the STRUCTURE software, which lacks a specific encoding to account for indels. This mechanism enables the program to preserve information on where the indel events occurred in the original alignment.
  - The concatenate_results function is called to concatenate the results, generating a string with the converted and filled sequences.
6. Generation of Structure file and results visualization
  The string generated by the concatenate_results function is used to create an output file in Structure format, containing the concatenated and filled sequences.
  - The graphical interface is updated with the generated string, using a scrolling text box (preview_textbox), allowing the user to preview the results before saving them as a file.
7. Logging
  - The software logs key events during its operation, including the selection and processing of files, the identification of variable sites, and any exceptions that are raised.
  - The log records include the logger's name, the logging level of the event, and the message describing the event.
  - The log messages are written to a file named “log.log” in the same directory as the script.
  - The logging level is set to INFO, which means that events of levels INFO, WARNING, ERROR, and CRITICAL will be tracked.

By following these steps, the program offers an efficient and reliable solution for converting multiple aligned FASTA files into a concatenated binary format file suitable for use with STRUCTURE software. To assess the effectiveness of this conversion tool, two chloroplast genes (trnD-trnT and trnH-trnK) and one nuclear gene (ITS) available on the internet were utilized for two Avicennia species (*Avicennia germinans* and *Avicennia schaueriana*) [10]

## Results and discussion

**Fig. 1:**
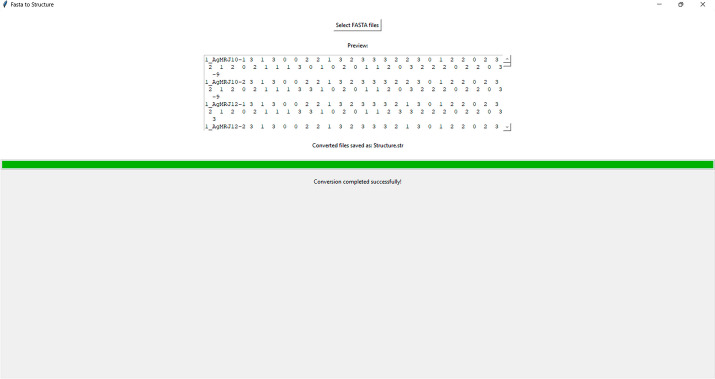
Graphical user interface of the software tool, showcasing the streamlined process of converting multiple aligned FASTA files into a single concatenated binary format file compatible with STRUCTURE software. The intuitive design allows for easy navigation and reduced error risk, making it accessible for researchers with varying programming experience

The tool streamlines the process of converting multiple aligned FASTA files into a single concatenated binary format file, suitable for use with the STRUCTURE software. Its user-friendly graphical interface simplifies data preparation and minimizes the risk of errors, making it accessible for researchers with limited programming experience (Fig. 1). The tool’s functionality was tested using various aligned FASTA files containing DNA sequence data from two species and multiple populations. The software consistently identified variable sites converted the sequences to structure format concatenated the binary sequences and generated a file in the STRUCTURE format.

**Fig. 2:**
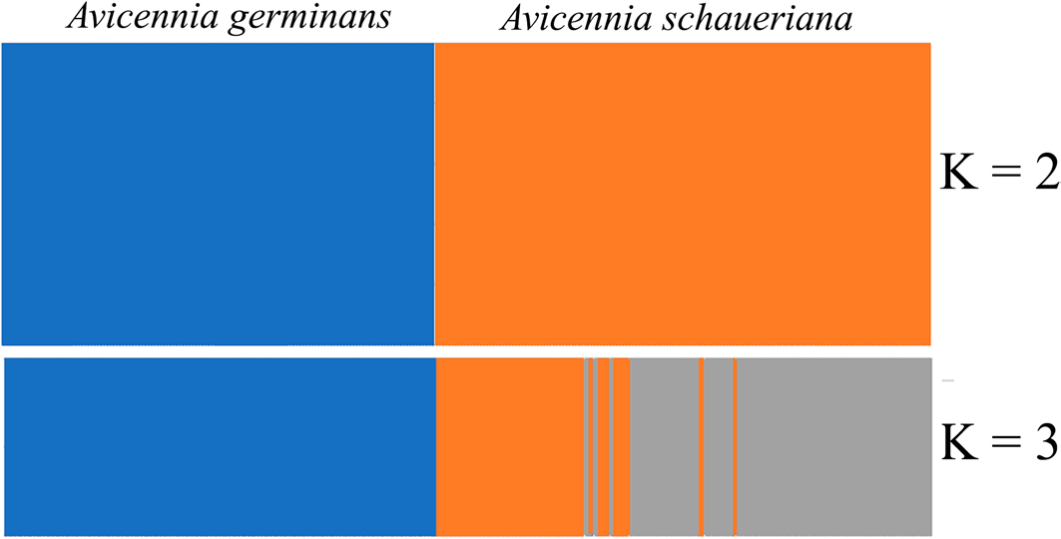
Graph showing the population structure analysis performed with the STRUCTURE software, using sequence data from two chloroplast genes and one nuclear gene from *Avicennia germinans* and *Avicennia schaueriana*. The simulations were conducted to evaluate the efficiency of data conversion using the tool developed in this study

During the evaluation phase, the performance of the software tool was assessed in terms of processing time and output quality. For small datasets, the software rapidly processed the FASTA files and generated the concatenated binary format file within seconds. However, processing times for larger datasets, containing more sequences and loci, varied depending on the computer’s processing capabilities. In our simulations and using publicly available data, the converted datasets demonstrated consistent detection of genetic variation by STRUCTURE at both population and species levels (Fig. 2). This consistency was evident through successful population structure analyses, highlighting the software’s reliability and accuracy in preparing data for STRUCTURE-based evaluations.

Additionally, the software adeptly managed missing data and varying sequence lengths by padding sequences with − 9 as needed to generate equal-length concatenated sequences. This essential feature guarantees the compatibility of the output files with the STRUCTURE software, accommodating real-world datasets that may have incomplete or inconsistent information. By maintaining the integrity of the data, the software ensures reliable and accurate population structure analyses, even when dealing with incomplete or variable-length data.

Overall, the developed software tool demonstrates robust performance and reliability in converting multiple aligned FASTA files to the STRUCTURE format. It has the potential to save researchers valuable time and effort in preparing their data for population structure analysis, facilitating a more efficient and error-free process. The developed software tool addresses a critical need in the field of population genetics, as the analysis of population structure often requires the conversion of multiple aligned FASTA files to a format compatible with the STRUCTURE software. By providing a user-friendly graphical interface and a robust, efficient conversion process, our software tool simplifies data preparation, enabling researchers to focus on the interpretation and application of their results.

While pre-existing tools with capabilities like those offered by fasta2structure do exist (Table 1), they often require users to have a robust understanding of bioinformatics, or their functionalities don’t exactly match those provided by fasta2structure. For instance, the Python program named “Convert-fasta-alignments-to-Structure-format” operates through the command line and necessitates that users specify input and output directories as arguments. Furthermore, this tool selects a single SNP (Single Nucleotide Polymorphism) from each input file, i.e., it generates one SNP per alignment, rather than converting all relevant variations from the complete sequence. This feature may be more useful for population genetic studies that need a subset of SNPs derived from data such as UCEs (Ultraconserved Elements) and exons, rather than full sequences.

**Table 1:** Comparative feature analysis of Fasta2Structure against other available codes

Feature	Fasta2Structure	Convert-fasta-alignments-to-structure-format	R script
Graphical user interface (GUI)	✓	✕	✕
Multiple file selection	✓	✕	✕
Reads files one by one	✓	✕	✓
Explicit error handling	✓	✕	✕
Searches for variable sites in each alignment	✓	✕	?
Uses threading	✓	✕	✕
Results visualization in the interface	✓	✕	✕
Stores results in a single file	✓	✓	✕

Presence (✓)

Absence (✕) or uncertainty

(?) Of a particular feature in the respective programs

Conversely, fasta2structure incorporates all variable sites present in the alignments, which may lead to a more accurate representation of the genetic variation embodied in the data. In this context, we deem fasta2structure to exhibit a higher degree of robustness in converting a wider array of data types, encompassing those with significant genetic variation. This characteristic is integral for in-depth studies in population genetics and phylogeography.

Another existing tool is provided in the form of an R script that uses the 'ape' library for data conversion. This tool demands a higher level of bioinformatics proficiency from the user as it necessitates script editing to adapt it to each user’s specific data. Additionally, the script provides guidelines assuming the user is operating on Ubuntu Linux version 20.04 as their operating system, thus requiring a virtual machine within a Windows or Mac PC to enable its usage on these platforms. This is in stark contrast with ‘fasta2structure’, which is universally compatible across any operating system. This compatibility can broaden its accessibility, making it an advantageous option for diverse users in the field of bioinformatics.

It’s worth noting that the R script codes are divided into two distinct scripts to process diploid and haploid data separately, unlike ‘fasta2structure’, which can interpret any degree of ploidy of interest to the user. Additionally, thanks to its multithreaded implementation, ‘fasta2structure’ can process FASTA files asynchronously, which can significantly enhance efficiency and processing time when dealing with large data sets. In contrast, the R script operates sequentially, a feature that may result in reduced speed when processing large volumes of data.

Fasta2structure is presented as an intuitive and user-friendly tool, both through its Graphical User Interface (GUI) and its efficient logging functionality. This logging is accomplished through a log file that synthesizes crucial information from the input files, such as the position of variable sites. In addition, the log file can provide valuable guidance to users for identifying and rectifying potential errors in alignments. Therefore, fasta2structure not only offers accessible data conversion but also supports users in troubleshooting and data quality assurance.

The importance of this tool is further highlighted by the current capacity to generate sequencing data for multiple genes simultaneously. As next-generation sequencing technologies continue to advance [11, 12], researchers are now able to obtain vast amounts of genetic information at an unprecedented scale. This increased capacity necessitates efficient tools for processing and analyzing such data, especially when studying population genetics and phylogenetics. By facilitating the seamless conversion of multiple aligned FASTA files into a concatenated binary format compatible with the STRUCTURE software, this tool greatly simplifies the data processing workflow and allows researchers to focus on the interpretation of their results [5, 9].

The use of Tkinter and Biopython libraries ensures that our software tool is accessible to a wide range of users, regardless of their programming experience. Tkinter allows for the creation of an intuitive graphical interface, while Biopython streamlines the processing and manipulation of FASTA files [13]. The combination of these libraries, along with the provided helper functions, allows for a seamless conversion process, minimizing errors and enhancing the overall user experience.

## Conclusions

The software tool developed in this study addresses a key challenge in population genetics research by providing an efficient and user-friendly solution for converting multiple aligned FASTA files to the STRUCTURE format [5]. Utilizing the capabilities of Tkinter [14] and Biopython [15] libraries, the software tool streamlines the data preparation process and accommodates various datasets, including those with missing or variable-length data.

Through testing and evaluation, the software tool demonstrated robust performance, reliability, and compatibility with the STRUCTURE software. The user-friendly graphical interface and efficient conversion process not only simplify data preparation but also reduce the likelihood of errors, making the tool accessible to a wide range of users.

In conclusion, the developed software tool offers an efficient and reliable solution for converting multiple aligned FASTA files to the STRUCTURE format [5]. It simplifies the data preparation process and accommodates missing or variable-length data, promoting more accurate and efficient population structure analysis. As the field of population genetics continues to evolve [16], we anticipate that tools such as this will play an increasingly important role in streamlining research workflows and facilitating scientific discovery.

## Data Availability

The data sets generated and/or analyzed during the current study are available in the: https://github.com/AdamBessa/Fasta2Structure. Software name: Fasta2Structure Software home page: https://github.com/AdamBessa/Fasta2Structure.git. Operating system(s): Linux, Mac, Windows. Programming language: Python 3.5 or higher. Dependencies: Tkinter, Biopython. License: MIT license. Any restrictions to use by non-academics: NONE.
